# Accelerated Aging Effect on Mechanical Properties of Common 3D-Printing Polymers

**DOI:** 10.3390/polym13234132

**Published:** 2021-11-26

**Authors:** Catalin Gheorghe Amza, Aurelian Zapciu, Florin Baciu, Mihai Ion Vasile, Adrian Ionut Nicoara

**Affiliations:** 1 Department of Quality Engineering and Industrial Technologies, Faculty of Industrial Engineering and Robotics, University “Politehnica” of Bucharest, 060042 Bucharest, Romania; 2 Department of Robotics and Production Systems, Faculty of Industrial Engineering and Robotics, University “Politehnica” of Bucharest, 060042 Bucharest, Romania; aurelianzapciu@yahoo.com; 3 Department of Strength Materials, Faculty of Industrial Engineering and Robotics, University “Politehnica” of Bucharest, 060042 Bucharest, Romania; florin.baciu@upb.ro (F.B.); vasileionmihai@yahoo.com (M.I.V.); 4 Department of Science and Engineering of Oxide Materials and Nanomaterials, Faculty of Applied Chemistry and Materials Science, University “Politehnica” of Bucharest, 060042 Bucharest, Romania; adrian.nicoara@upb.ro

**Keywords:** 3D printing, PLA, PETG, accelerated aging, ultraviolet

## Abstract

In outdoor environments, the action of the Sun through its ultraviolet radiation has a degrading effect on most materials, with polymers being among those affected. In the past few years, 3D printing has seen an increased usage in fabricating parts for functional applications, including parts destined for outdoor use. This paper analyzes the effect of accelerated aging through prolonged exposure to UV-B on the mechanical properties of parts 3D printed from the commonly used polymers polylactic acid (PLA) and polyethylene terephthalate–glycol (PETG). Samples 3D printed from these materials went through a dry 24 h UV-B exposure aging treatment and were then tested against a control group for changes in mechanical properties. Both the tensile and compressive strengths were determined, as well as changes in material creep characteristics. After irradiation, PLA and PETG parts saw significant decreases in both tensile strength (PLA: −5.3%; PETG: −36%) and compression strength (PLA: −6.3%; PETG: −38.3%). Part stiffness did not change significantly following the UV-B exposure and creep behavior was closely connected to the decrease in mechanical properties. A scanning electron microscopy (SEM) fractographic analysis was carried out to better understand the failure mechanism and material structural changes in tensile loaded, accelerated aged parts.

## 1. Introduction

The 2020 COVID-19 pandemic has illustrated how easily global supply chains can become stressed, leading to a shortage of manufactured goods [1] and raw materials, either through a slowdown in production [2] or through delays in transportation [3]. Reports such as a study by Deloitte on business leaders in the United Kingdom say that the current supply issues encountered in some sectors might extend until 2023 even without further pandemic-related restrictions [4]. In an editorial article, Flynn et al. analyzed several essays concerning supply management in disruptive times and made a call for new insights, opportunities, and research questions [5]. It is worth noting that such supply chain disruptions are expected to become more frequent, as a result of environmental and climate changes [6,7,8]. These signals show that decentralized supply chains, such as those enabled by 3D printing [9,10,11], could help alleviate future disruptions.

3D printing, a manufacturing process that builds parts layer by layer, has seen a widespread adoption in the past few decades [12]. Starting in 2009, with the expiration of patents surrounding Fused Deposition Modeling (FDM) [13], a process that uses extruded thermoplastic polymers as feedstock, this fabrication method has seen a rapid expansion in adoption by home users [14], public facilities and small- and medium-sized businesses [15]. This rapid expansion was enabled by advances made in machine control technology together with the open-sourcing of mechanical, hardware and software designs [16,17,18,19,20]. According to a yearly survey conducted by Sculpteo (Villejuif, France), in 2020, Material Extrusion (MEX/FDM) was the 3D-printing process with the most widespread usage among professionals from 71 different countries [21]. The same report highlights a trend of increased usage of 3D printing to manufacture functional, end-use parts, with over 50% of the questioned professionals leveraging 3D printing for this use case.

In the most common implementation of MEX, a thermoplastic filament feedstock is pushed through a heated nozzle and extruded in thin filaments that are deposited on a build plate, forming a horizontal layer of an object. Subsequently, the nozzle position is incremented and a new layer of material can be deposited on top of previously deposited layers. Some of the most commonly used materials with MEX 3D printing include acrylonitrile butadiene styrene (ABS) [22], polylactic acid (PLA) [23,24], polyethylene terephthalate glycol-modified (PETG) [25,26] and nylons [27,28]. Among these, PLA and PETG distinguish themselves as being easy to process and relatively inexpensive, while also requiring inexpensive 3D-printing equipment.

Environmental conditions can affect polymers used outdoors in a multitude of ways. Exposure to chemicals, UV radiation, and temperature cycles can cause the depolymerization, chemical degradation or photodegradation of polymers [29,30,31].

The literature contains extensive studies on the aging of polymers in atmospheric conditions [32,33]. Among these studies, the accelerated aging of PLA blends has also been investigated, highlighting the reduction in mechanical properties following prolonged ultraviolet exposure [34,35]. However, accelerated aging effects on 3D-printed parts made from these materials has been studied more sparsely, given the relatively new and innovative use cases. Other than atmospheric effects, chemical effects have also been analyzed. For example, in the context of the COVID-19 pandemic, Grzelak et al. analyzed the effects of alcohol disinfection on ABS and PETG parts meant for use in a medical setting and concluded that PETG parts can lose up to 20% of their tensile properties after repeated sterilization [36]. Moreno Nieto et al. investigated the design of parts made from PLA and PETG for water environments and found that PLA degrades significantly more than PETG due to its organic nature [37]. More so, Cuiffo et al. found that 3D printing rearranges the molecular polymeric chains of PLA and increases its water susceptibility compared to other manufacturing techniques [38].

In parts made to sustain prolonged stress, material creep also becomes an important property to consider. Martins et al. researched the short-term creep behavior of PLA-PCL blends and found that most of the deformation occurs right after loading, after which the creep rate decreases with time [39]. Morreales et al. highlighted how the addition of fibers changes the creep compliance of PLA biopolymers [40], while Shanmugam et al. provided insight as to why fatigue testing is difficult to determine in additively manufactured parts due to the layered aspect of the process [41].

In this context, this paper intends to provide more insight on the changes in the mechanical properties suffered by 3D-printed parts made from PLA and PETG after prolonged exposure to the Sun. By simulating prolonged exposure with a controlled irradiation treatment, the same damaging effect can be inflicted in a much shorter time. Among the properties investigated in this paper are tensile and compression strength, part stiffness and tensile and compression creep behavior changes.

## 2. Materials and Methods

UV radiation reaching the Earth’s surface is just a small percentage of total solar irradiance [42]. In the stratosphere, the ozone layer absorbs the shortest wavelengths (UV-C, 100–280 nm) and weakly absorbs wavelengths in the UV-B range (280–315 nm). Most of the UV-A wavelengths (315–400 nm) pass through the stratosphere unabsorbed and reach the Earth’s surface [43]. As a result of sunlight path, altitude and atmospheric conditions, UV-A makes up 94% of UV energy at ground level while UV-B accounts for the remaining 6% [44].

A method to experimentally replicate the effect of weather and Sun exposure on polymers involves irradiating test samples with UV-A or UV-B radiation in an irradiation chamber and is detailed in standard ISO 4892-3:2016 [45].

For the purpose of analyzing the effects of accelerated aging on the mechanical properties of 3D-printed parts, samples made from PLA and PETG were 3D printed on a Creality Ender-3 3D printer (Shenzhen Creality 3D Technology, Shenzhen, China). Blue (opaque) PLA filament was procured from Fillamentum Manufacturing Czech (Hulin, Czech Republic) under the brand name Fillamentum PLA Extrafill. The filament is 1.75 mm in diameter and the manufacturer specifies a glass transition temperature of 55 °C and a melting temperature of 145–160 °C. Natural color (transparent) PETG filament, 1.75 mm in diameter, with a glass transition temperature of 86 °C and a melting temperature of 255 °C was sourced from Prima Printer Nordic AB (Malmö, Sweden) under the brand name PrimaSelect. Printing process parameters are shown in Table 1. Printed parts were sliced in Cura version 4.5 slicer (Ultimaker, Utrecht, The Netherlands). The printing process took place at an ambient temperature of 23° C and 50% humidity.

**Figure 1:**
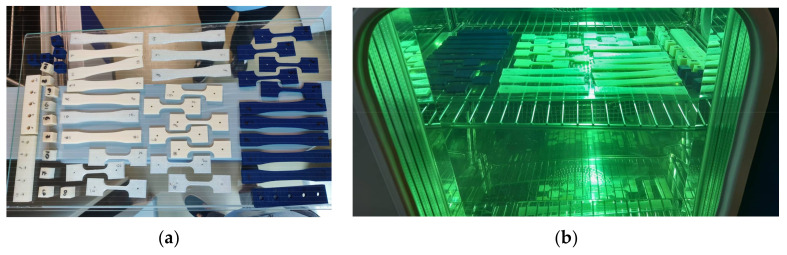
Tested materials: (**a**) 3D-printed parts; (**b**) UV-B exposure of tested group.

3D-printed parts were split into control and treatment groups, consisting of 5 samples each, for each type of material (PLA, PETG) and each type of planned destructive test (tensile strength, compressive strength, tensile creep). Four samples were manufactured for compression creep testing. Thus, a total of 64 samples were manufactured and then analyzed (Figure 1a).

Parts in the treatment group were subjected to UV-B irradiation, 310 nm wavelength. A Discovery DY110C (ACS) thermostatic climate chamber was used to experimentally determine the influence of UV radiation. The treatment consisted of cycles of 8 h dry treatment with lamps at 0.43 Wm^−2^ × nm^−1^ followed by 4 h condensation with UV lamps turned off (Figure 1b). The dry cycles were performed at 50°C and 50% ambient humidity. Both parameters were set using the control unit of the chamber. During the condensation cycle, parts were left to cool to 23 °C while humidity was maintained at 50%. The maximum deviation allowed for ambient humidity between cycles was 10% of the set value. Total UV exposure time was 24 h (3 cycles). The length of the total exposure was chosen considering the average solar UV-B radiation that reaches the surface of the Earth of 0.25 Wm^−2^ [46,47]. Thus, the total material UV exposure in the chamber is equivalent to several months of outdoor exposure to the Sun. This is an approximation, as the effects of the shorter wavelength UV-B radiation on materials and organic molecules are more extensive than those of UV-A radiation [30,48,49].

Destructive tensile strength tests were performed on 10 dog-bone samples from each material sized according to ASTM D638-14 Type I dimensions [50].

Each group consisted of 5 samples manufactured with the same set of parameters. All samples were tested in standard atmospheric conditions, 23 °C and 50% humidity, as specified by standard ISO 692 [51]. The same atmospheric conditions were maintained for all mechanical tests. Tensile strength testing was conducted on a universal testing machine Instron 8872 (Instron, Norwood, MA, USA), with a preload value of 5 N and a loading speed of 1 mm / minute. An electronic extensometer was used to measure sample elongation during traction. Results of tensile strength testing are shown in Section 3.2.

**Figure 2:**
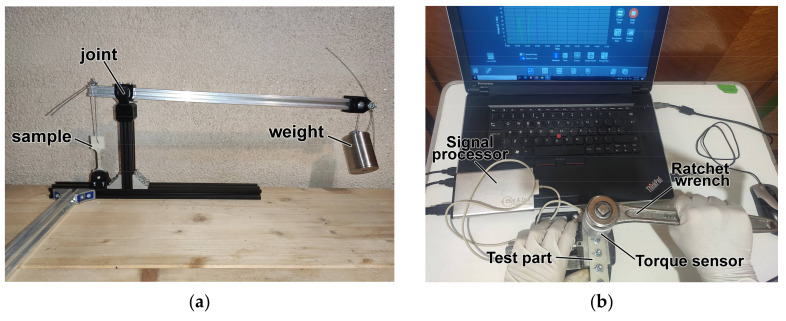
Testing of creep properties: (**a**) tension creep testing rig; (**b**) compression creep testing setup.

For destructive compression strength tests, 10 cubic samples of 15 × 15 × 15 mm in size were printed from each of the 2 materials and tested on an Instron 8801 machine (Instron, Norwood, MA, USA). Parts were loaded with a speed of 1 mm/min along the *Z*-axis starting with a preload force of 5 N. Results of compressive strength testing are shown in Section 3.3. Images of the two mechanical testing setups described above are available in Supplementary Information (Figures S1 and S2). Following tensile and compressive testing, the creep characteristics of the materials were also assessed. Creep is the property of certain materials to deform plastically over time under loads significantly lower than the loads allowed by ultimate strength. Ten samples were 3D-printed from each of the two materials according to dimensions found in ASTM D2990-17 [52]. A testing rig was designed to load parts in tension (Figure 2a). Samples were loaded with approximately 25% of the load found at ultimate strength for the control group for each material. The distance between the sample ends were measured using a micrometer after 2 h, 6 h, 12 h and 24 h, followed by measurements every 24 h for a total load time of 1 week. Sample elongation was calculated based on the average between the two measurements and elongation vs. time charts were drawn. Additionally, two samples from each material were printed to assess compressive creep. Each sample had 5 bores 6 mm in diameter where a M6 nut and bolt assembly was mounted. The nut and bolt assembly was tightened using a torque wrench (Figure 2b) and the input torque was measured using a MR-55 1000 torque sensor made by Mark-10 Corporation (Copiague, New York, NY, USA). The clamping force was delivered to the 3D-printed material through two washers on each side of the material, with an external diameter of 12 mm and an internal diameter of 7.5 mm. Images of samples 3D printed for creep tests are available in Supplementary Information (Figures S3–S5).

The microstructures of the studied materials were assessed using a Quanta Inspect F50 scanning electron microscope (1.2 nm resolution—Thermo Fisher—formerly FEI—Eindhoven, The Netherlands) (SEM-EDS). One sample from each group (control PLA, UV-B PLA; control PETG, UV-B PETG) was coated with Au for 90 s and analyzed at different magnifications in vacuum using the electron beam accelerated at 20 KV with spot 5. Results of this analysis are shown in Section 3.5.

## 3. Results

### 3.1. Visual and Dimensional Inspection

**Figure 3:**
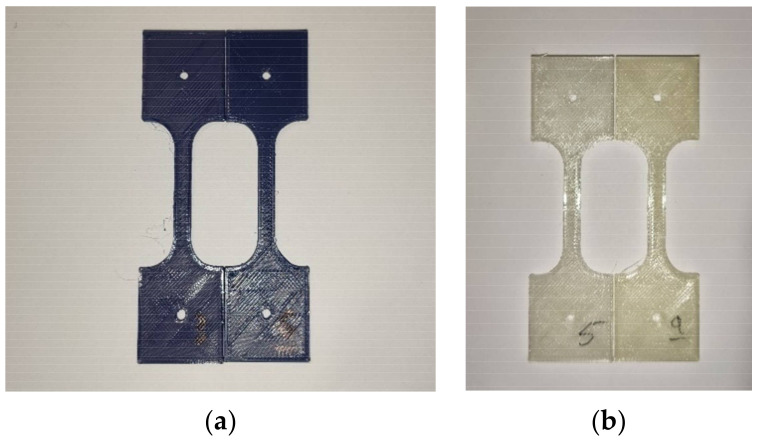
Changes in material color and reflectivity, (**a**) PLA part comparison between control group (left) and treatment group (right); (**b**) PETG part comparison, control group (left) and treatment group (right).

After irradiating the sample parts, a series of visual and dimensional measurements were performed. The accelerated aging process did not have any statistically significant effect on the dimensions of the inspected parts. This finding is true for both thin and long parts and for bulky parts, regardless of the printing orientation. The results from dimensional measurements in the control and tested groups for the different types of 3D-printed parts analyzed in this study are available in Supplementary Information (Table S1). A visual inspection of the parts showed some changes that occurred after the radiation treatment. For PLA (blue, opaque), the UV-B treatment slightly darkened and increased the shine of investigated samples (Figure 3a). Samples made from PETG (natural, transparent) gained a yellow tint and became darker following treatment (Figure 3b).

### 3.2. Tensile Strength and Stiffness

**Figure 4:**
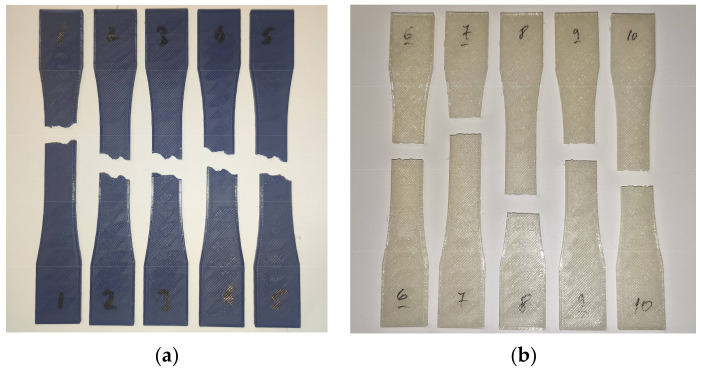
Fracture modes of tested parts: (**a**) PLA parts from the control group; (**b**) PETG parts from the UV-B exposure group.

The samples made from PLA fractured in a similar manner in both the control and UV-B treated groups. The fracture occurred along the deposited filament layers and formed a zig-zag pattern (Figure 4a). A low amount of part whitening due to stress can be observed near the failure point. Samples made from PETG ruptured along a more consistent plane perpendicular to the load, indicating superior interlayer adhesion (Figure 4b). The same rupture pattern was exhibited by parts in both the control group and the UV-B group, indicating that, while substantial, the changes in mechanical properties were sufficiently uniform to not cause a different failure mode.

**Figure 5:**
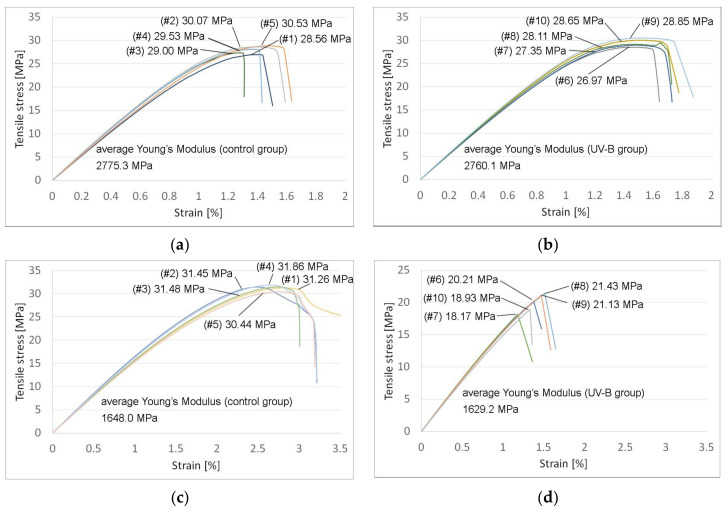
Tensile strength testing results, with Young’s Modulus and tensile stress at tensile strength: (**a**) PLA control group; (**b**) PLA with UV-B exposure; (**c**) PETG control group; (**d**) PETG with UV-B exposure.

Figure 5a–d show the stress–strain diagrams obtained from tensile testing.

Parts made from PLA showed a 5.3% loss in tensile strength following UV-B exposure compared to those in the control group. The stiffness of the irradiated parts saw an insignificant change (2760 MPa vs. 2775 MPa).

UV-B exposure also weakened the samples made from PETG, which showed a significant 36% loss in tensile strength compared to those in the control group. The stiffness of the irradiated PETG parts did not change significantly (1629 MPa vs. 1648 MPa). Table 2 shows the average tensile strength and the Young’s Modulus of the tested parts with standard error.

### 3.3. Compressive Strength

**Figure 6:**
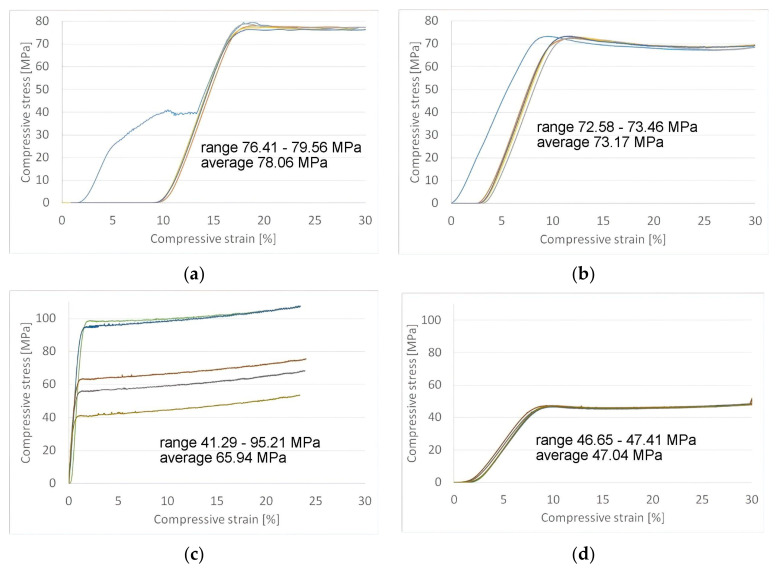
Compressive strength testing results: (**a**) PLA control group; (**b**) PLA with UV–B exposure; (**c**) PETG control group; (**d**) PETG with UV–B exposure.

Samples made from PLA and PETG failed plastically under compression load without any cross-sectional shear visible in the outer perimeter of the parts. For PLA, irradiated parts were weaker, losing 6.3% (73.17 MPa vs. 78.06 MPa) of the compressive strength for the control group. The same effect was observed for PETG parts, where the compressive strength for the UV-B-treated group was 38.6% lower than in the control group (47.04 MPa vs. 65.94 MPa). Figure 6a–d show the stress–strain diagrams obtained following compressive testing.

The average compressive strength of the tested parts with standard errors is compiled in Table 3.

### 3.4. Creep Testing

As mentioned previously, the loads for tensile creep testing were selected based on the ultimate strength values found for the control groups during tensile testing. For the tensile creep test, the testing rig has a variable mechanical advantage that increases the load applied at one end by up to 10-fold. The applied load was calculated considering that the tested specimens have a cross section of 5.2 mm × 3 mm, resulting in a cross-section area of 15.6 mm^2^.

For compressive creep testing, the force was applied onto the material using the washer with a 12 mm outer diameter and a 7.5 mm inner diameter, resulting in a cross section of 62.92 mm^2^. The tightening torque for the screw was chosen based on the standard tightening torque for an M6 screw. The compression force can be calculated considering a screw diameter D = 6 mm, a screw torque coefficient K = 0.3 and the tightening torque. The loads used during this experiment are shown in Table 4.

**Figure 7:**
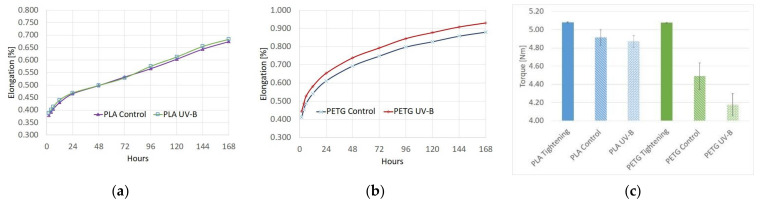
Creep curves of tested materials: (**a**) PLA elongation vs. time; (**b**) PETG elongation vs. time; (**c**) compression creep test untightening torque results.

Figure 7a shows a graph of the elongation of the PLA parts under load over the 7-day (168 h) testing period, while Figure 7b shows the same graph for PETG. For both materials, most of the deformation occurred in the initial moments after stressing the test parts and the creep rate decreased as the strain level was increased. As can be seen in the graphs, the parts exposed to UV-B deformed more under the load but displayed a similar creep curve pattern. The heavier deformation is thus considered to occur due to the weakening of the parts. In terms of compression creep behavior, all investigated samples untightened at a torque lower than the standard tightening torque of 5.07 N·m after 7 days. For the control group PLA samples, the clamping force was reduced by 3.3% to 4.92 ± 0.04 N·m, while the control PETG samples saw a bigger reduction, with the untightening torque being reduced by 11.5% to 4.49 ± 0.07 N·m. In the PLA samples that were subjected to UV-B radiation, the untightening torque was virtually unchanged compared to the control group, with the screws requiring on average 4.87 ± 0.04 N·m of torque to untighten. The PETG samples in the UV-B group, however, saw a 7.5% further decrease in torque compared to the control group, with the average required torque being 4.18 ± 0.06 N·m. A bar chart of the experimental results for the untightening torque measurements is shown in Figure 7c.

### 3.5. Scanning Electron Microscopy (SEM)

**Figure 8:**
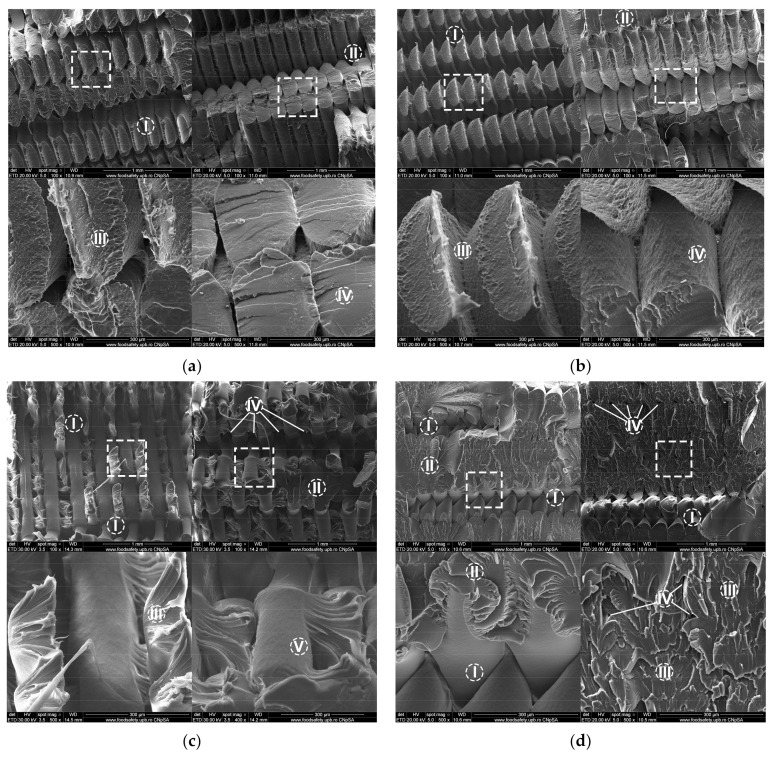
Scanning electron microscopy analysis of fractured 3D-printed samples: (**a**) PLA sample from control group: I—fracture along filament lines; II—filaments fractured transversally; III—sharp necking of the ruptured filaments; IV—reduced flaking at the fractured surface; (**b**) PLA sample from UV-B-exposed group: I—fracture along filament lines; II—filaments fractured transversally; III—sharp necking of the ruptured filaments; IV—increased roughness of filaments surface; (**c**) PETG sample from control group: I—fracture along filament lines; II—filaments fractured transversally; III—flaking and necking of the ruptured filament; IV—internal microvoids specific to the manufacturing process; V—interlayer fusion; (**d**) PETG sample from UV-B-exposed group: I—fracture along filament lines; II—filaments fractured transversally; III—flaking of the ruptured filaments; IV—internal microvoids specific to the manufacturing process.

Fractographic analysis using SEM was carried out to gain better understanding of the failure mechanism for tensile loaded samples. Figure 8a shows a SEM image taken of a PLA part belonging to the control group, while one taken of a part belonging to the irradiated group is shown in Figure 8b. A SEM image taken of a PETG part belonging to the control group is shown in Figure 8c, while an UV-B exposed part is shown in Figure 8d.

The PLA samples ruptured along the deposited filament lines. Sharp necking of the filaments at the fracture interface was found in the control sample (Figure 8aIII), but an increase in this phenomenon can be seen in the irradiated sample (Figure 8bIII). An increase in surface roughness can also be seen in the irradiated PLA specimen (Figure 8bIV).

Changes in the PETG sample microstructure following UV-B treatment are more obvious. The irradiated sample has a more extensive area where filaments fractured transversally (Figure 8dII), compared to the area where the fracture occurred along filament lines (Figure 8dI). Due to the layered aspect of MEX and the alternate 45°/−45° raster directions used to deposit the material, triangular microvoids formed in the parts’ structures. These microvoids, specific to the 3D-printing process [53], can be seen in both samples at the fracture interface and did not change significantly following aging through UV exposure. Interlayer fusion is clearly visible in the control part (Figure 8cV) and less so in the irradiated part, where flaking of the ruptured surface is more present (Figure 8dIII).

## 4. Discussion

In the context of the ever-increasing usage of 3D printed parts in functional applications, it is important to assess whether design rules meant for injection molding or other classical manufacturing techniques also apply to 3D printing. This paper provides some insight into that issue by analyzing the effects of accelerated aging through UV-B exposure on the mechanical properties of parts made from PLA and PETG. Tensile and compressive tests were performed on groups of five specimens 3D printed from the two materials. Each group of UV-B-exposed parts was compared to an untreated control group in a series of mechanical strength and creep tests.

Following exposure to UV-B radiation, which simulates the effect of the Sun’s UV action, the tested parts darkened and saw an increase in reflectivity. No significant dimensional changes were observed post-treatment, a statement which is true for long and thin parts (tensile creep specimens, with a 5.2 mm × 3 mm section) and for bulky parts (compressive creep specimens, with a 25.2 mm × 8 mm section). SEM imaging revealed a change in the roughness at the surface of deposited filaments, indicating a potential depolymerization process. The irradiated groups saw a reduction in tensile and compressive strengths compared to the control groups. The reduction in tensile strength was 5.3% for PLA parts and 36% for PETG parts. A similar reduction was seen in the compressive strength, with 6.3% lower strength for PLA and 38.6% lower strength for PETG. Despite the big difference in mechanical properties, the fractographic imaging and SEM analysis of the PETG parts indicated similar failure modes for the treatment and control groups, indicating that the changes occurred uniformly in the part. It is worth investigating further if these uniform changes are specific to the material or if they happened due to the high transparency and the lack of added pigment. However, it is important to note that the PLA parts, even with the presence of an added blue pigment, had the same behavior as the PETG parts, although the reduction in mechanical strength was considerably smaller. The elastic properties of the two materials did not change significantly, as their Young’s Modulus was virtually unchanged following ultraviolet exposure.

Despite the use of standard test specimens for tensile strength testing, the findings presented in this paper should only be associated with the ASTM D638-14 Type I dimensions of the test part. According to research by Laureto et al., the type I dimensions will produce slightly better tensile strength results compared to type IV ones when used with a MEX 3D-printing process [54].

Tensile creep tests backed the findings regarding material stiffness as the elongation vs. time curves for both materials were similar for the tested and control groups, with the obvious offset due to the weakening of mechanical properties. For both materials, most of the deformation occurred within the first hours of stress loading. A practical test was performed in order to assess compression creep by mounting a screw and nut assembly on a 3D-printed part and tightening it to a standard torque. After 1 week of loading, the assembly was untightened and the required torque was measured. It was found that for PLA parts, the clamping force dropped by 3.3% in the specified timeframe. For PETG parts, the effect was more significant, with the clamping force reducing by 11.5% in the same timeframe. After UV-B treatment, the clamping force on the PLA parts was virtually identical to that found in the previously described control group. For PETG, there was a further reduction in clamping force, up to 21.3% less than the force resulting from the standard 5.07 N·m tightening torque. Given the anisotropic character of MEX 3D-printed parts, it is worth noting that these findings should only be associated with the 45°/−45° infill raster angle used when fabricating the samples. Zhang et al. studied the creep characteristics of ABS 3D-printed parts and found that infill orientation plays an important role in creep resistance, with a 90° orientation being the most creep-resistant [55]. These findings highlight the need of further testing with different process parameters.

## 5. Conclusions

This paper analyzed the effects of accelerated aging through UV-B exposure on the mechanical properties of 3D-printed parts made from PLA and PETG, two common MEX 3D-printing materials. Following mechanical testing, it was found that the mechanical strength decreased slightly for PLA samples undergoing a 24 h UV-B exposure and considerably for PETG samples undergoing the same treatment. The same tests found that UV-B radiation had an insignificant effect on part stiffness.

A tensile creep test revealed that creep characteristics are maintained following ultraviolet exposure and the two materials display similar creep curves, with the obvious offset caused by the weakening of the mechanical strength. A practical compression creep test was performed, where a screw and nut assembly was mounted on a 3D-printed part and tightened to a standard torque. After 1 week of tensioning, the assembly was untightened and the required torque was measured. It was found that material creep slightly reduces the clamping force on the PLA sample and substantially reduces it on the PETG sample. The reduction in clamping force remains the same for treated PLA parts but increases even further for treated PETG parts.

The exposure to outdoor sunlight reduces the mechanical properties of 3D-printed parts made from the investigated materials. It is thus mandatory for designers to consider the damaging effects of ultraviolet radiation on parts specifically made to function in direct sunlight. Extra attention needs to be given to parts that function under continuous stress and that will suffer from material creep. As with all 3D-printing tests, it is very important to note that feedstock material blends from different manufacturers, different 3D-printing machines and different printing parameters or software, may produce slightly different results, as highlighted in research conducted by Popescu et al. [56]. In order to correctly assess mechanical property changes following a specific treatment, we recommend in-house testing of the material and 3D printer combination before producing polymer parts destined for functional use.

This paper focused on determining the amount of change in mechanical properties of 3D-printed materials subjected to UV-B radiation. Future studies could focus on changes occurring at the molecular level. A careful design of such a study is necessary, in order to account for the variability in 3D-printing material feedstock. Additionally, such a study would be very sensitive to temperature conditions found in the 3D-printing process.

## Figures and Tables

**Table 1:** 3D-printing process parameters.

Material	Nozzle Diameter	Layer Height	Contours	Infill	Infill Pattern	Extrusion Temp.	Bed Temp.
PLA	0.40 mm	0.20 mm	2	100%	Grid 45°/−45°	205 °C	45 °C
PETG						235 °C	65 °C

**Table 2:** Tensile strength and stiffness.

Property	PLA Control	PLA UV-B	PETG Control	PETG UV-B
Tensile strength [MPa]	29.54 ± 0.35	27.99 ± 0.36	31.30 ± 0.24	19.98 ± 0.63
Young’s Modulus [MPa]	2775 ± 26.3	2760 ± 44	1648 ± 21.5	1629 ± 14.6
Elongation at break [%]	1.68 ± 0.02	1.43 ± 0.05	3.06 ± 0.04	1.36 ± 0.06

**Table 3:** Compressive strength.

Property	PLA (Control)	PLA (UV-B)	PETG (Control)	PETG (UV-B)
Compressive str.	78.06 ± 0.55	73.17 ± 0.17	65.94 ± 9.0	47.04 ± 0.16

**Table 4:** Loads used for creep testing.

Material	Tensile Properties				Compressive Properties			
	Strength [MPa]	Creep Test [MPa]	Target Load [N]	Used Load [N]	Strength [MPa]	Torque [N·m]	Used Load [N]	Creep Test [MPa]
PLA	29.54	7.5	117	12.5 × 9.4	78.06	5.07	2816	40.9
PETG	31.3	8	125	12.5 × 10	65.94	5.07	2816	40.9

## Data Availability

The data that support the findings of this study are available from the corresponding author upon reasonable request.

## Supplementary Materials

The following are available online at https://www.mdpi.com/article/10.3390/polym13234132/s1, Figure S1: 3D-printed part (dogbone) during tensile strength testing on Instron 8872 machine. Figure S2: 3D-printed part (cubic) during compressive strength testing on Instron 8801 machine. Figure S3: 3D-printed parts for tensile creep testing, from PLA. Figure S4: 3D-printed parts for tensile creep testing, fromPETG. Figure S5: 3D-printed parts for practical compressive creep test, from PLA (blue) and PETG (transparent, white). Table S1: Measured dimensions of 3D-printed parts from control group and group exposed to UV-B.
