# Supplementary material for: Accelerated Aging Effect on Mechanical Properties of Common 3D-Printing Polymers
Source: Polymers (Basel). 2021 Nov 26;13(23):4132. doi: 10.3390/polym13234132 (PMC8659210; doi:10.3390/polym13234132)
Supplement: Supplementary file 1 [file polymers-13-04132-s001.zip › polymers-1467727-supplementary.pdf]

polymers

Article

Accelerated Aging Effect on Mechanical Properties of Common 3D-Printing Polymers

Catalin Gheorghe Amza [1,*], Aurelian Zapciu[2], Florin Baciu[3], Mihai Ion Vasile[3] and Adrian Ionut Nicoara[4]

1 Department of QualityEngineering and Industrial Technologies, Faculty of Industrial Engineering and Robotics, University "Politehnica" of Bucharest, 060042 Bucharest, Romania; acata1@camis.pub.ro
2 Department of Robotics and Production Systems, Faculty of Industrial Engineering and Robotics, University "Politehnica" of Bucharest, 060042 Bucharest, Romania; aurelianzapciu@yahoo.com
3 Department of Strength Materials, Faculty of Industrial Engineering and Robotics, University "Politehnica" of Bucharest, 060042 Bucharest, Romania; florin.baciu@upb.ro (F.B.); vasileionmihai@yahoo.com (M.I.V.);
4 Department of Science and Engineering of Oxide Materials and Nanomaterials, Faculty of Applied Chemistry and Materials Science, University "Politehnica" of Bucharest, 060042 Bucharest, Romania; adrian.nicoara@upb.ro
* Correspondence: acata1@camis.pub.ro or amza_catalin@yahoo.com; Tel.: +40-744-500-803

Supplementary Materials

Citation: Amza, C.G.; Zapciu, A.; Baciu, F.; Vasile, M.I.; Nicoara, A.I. Ultraviolet Lamp Accelerated Aging Effect on Mechanical Properties of Common 3D-Printing Polymers. *Polymers* **2021**, *13*, 4132. https://doi.org/10.3390/polym13234132

Academic Editor: Giovanni Gómez-Gras

Received: 1 November 2021
Accepted: 23 November 2021
Published: 26 November 2021

Publisher's Note: MDPI stays neutral with regard to jurisdictional claims in published maps and institutional affiliations.

Copyright: © 2021 by the authors. Licensee MDPI, Basel, Switzerland. This article is an open access article distributed under the terms and conditions of the Creative Commons Attribution (CC BY) license (http://creativecommons.org/licenses/by/4.0/).

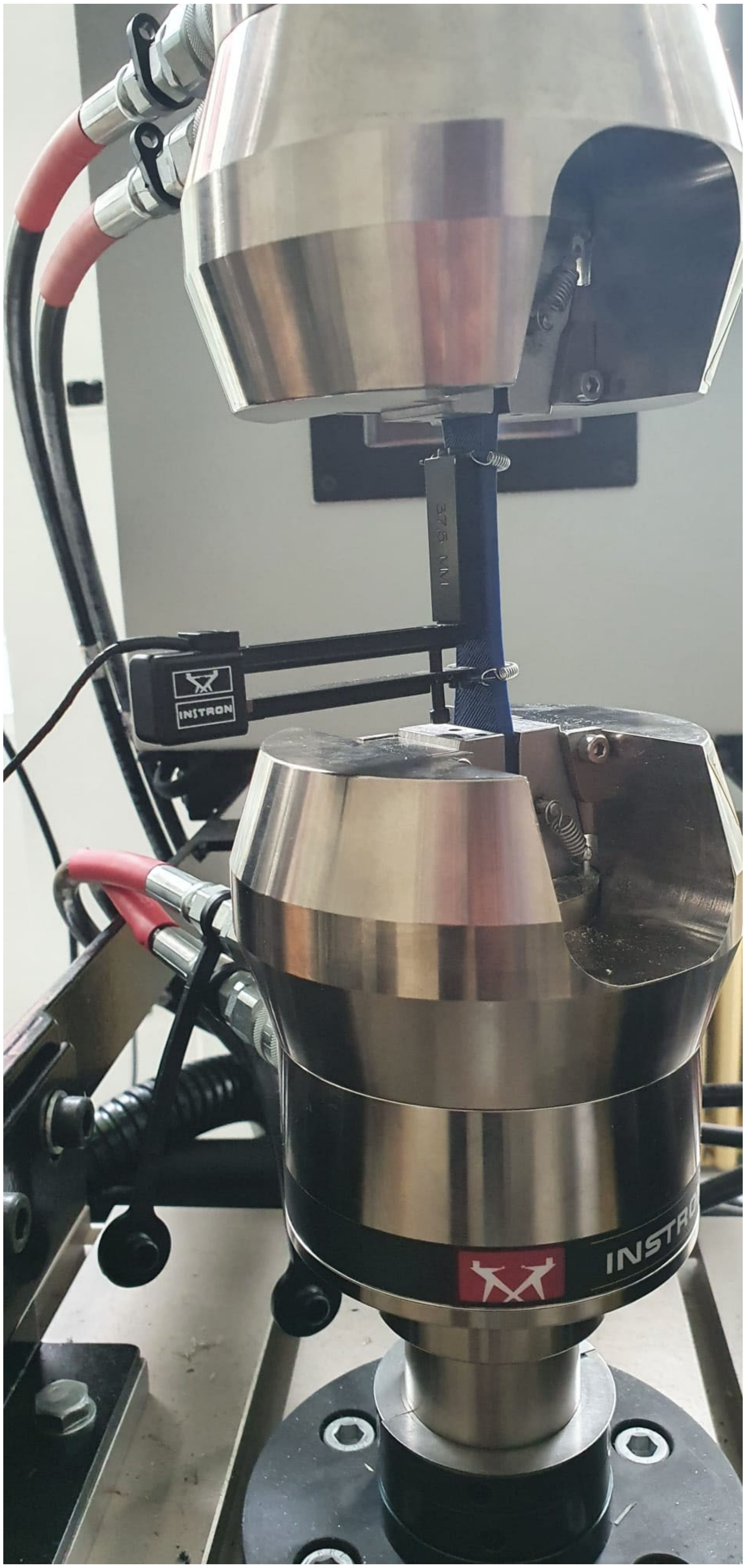

Figure S1. 3D-printed part (dogbone) during tensile strength testing on Instron 8872 machine.

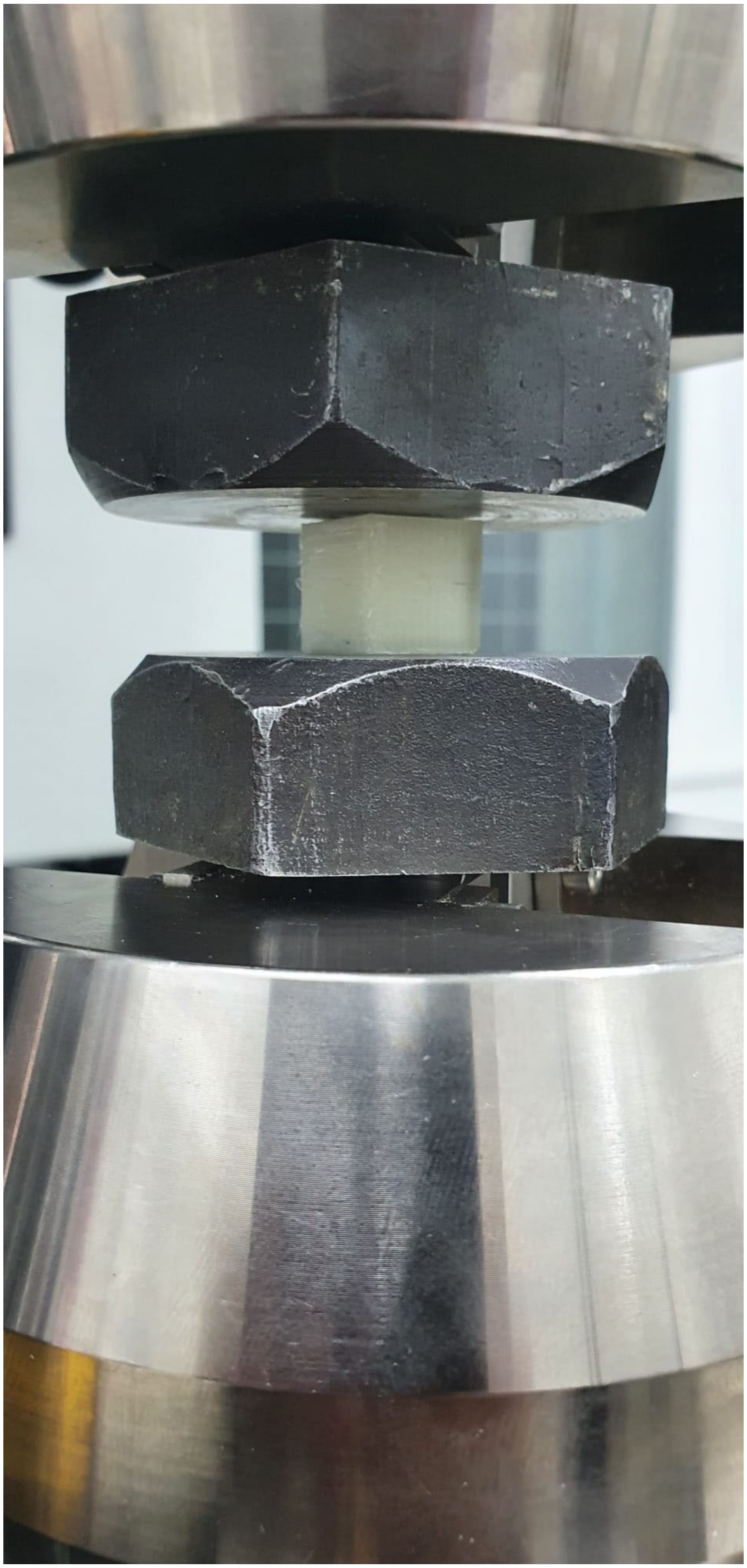

Figure S2. 3D-printed part (cubic) during compressive strength testing on Instron 8801 machine.

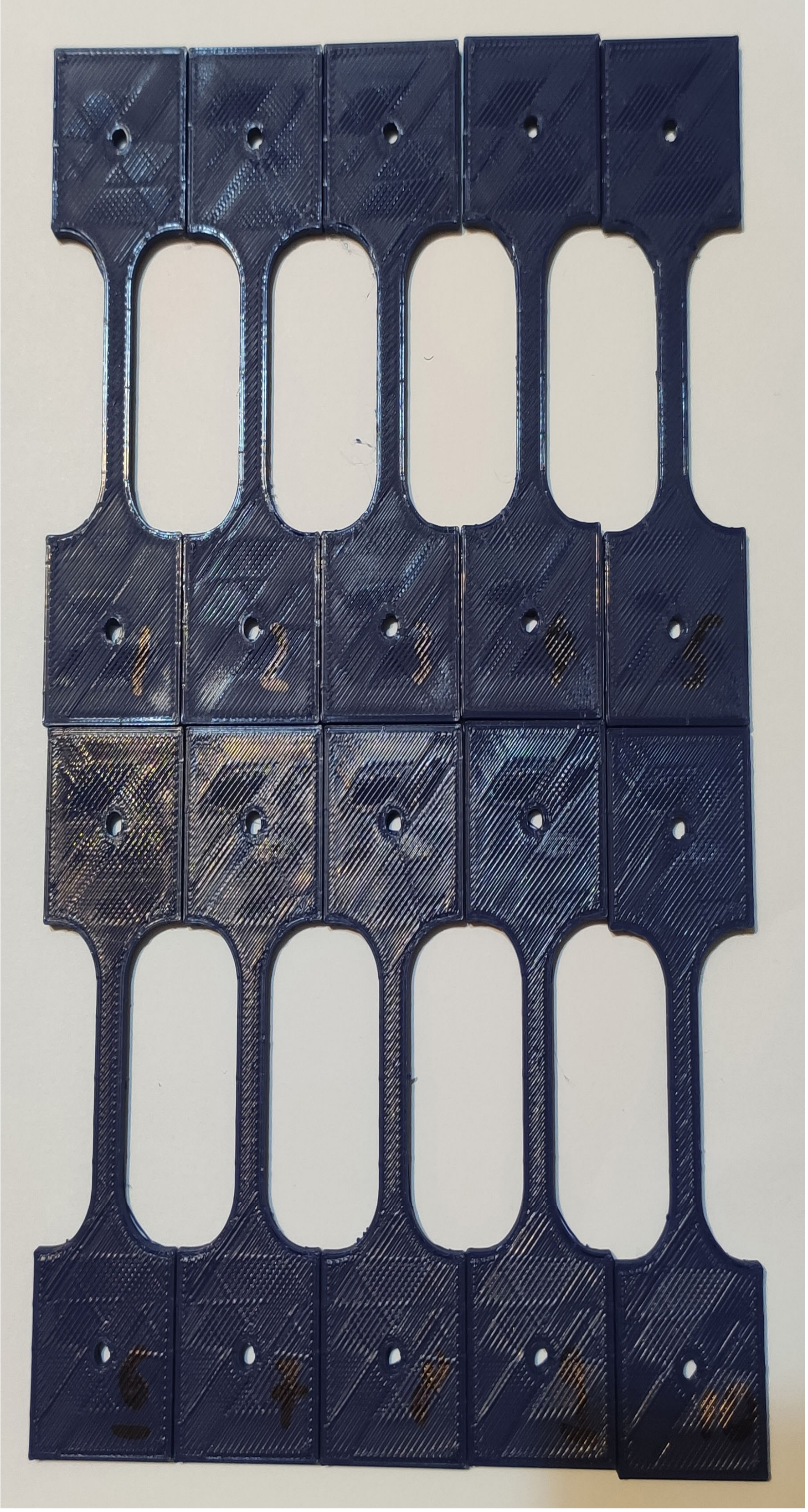

Figure S3. 3D-printed parts for tensile creep testing, from PLA.

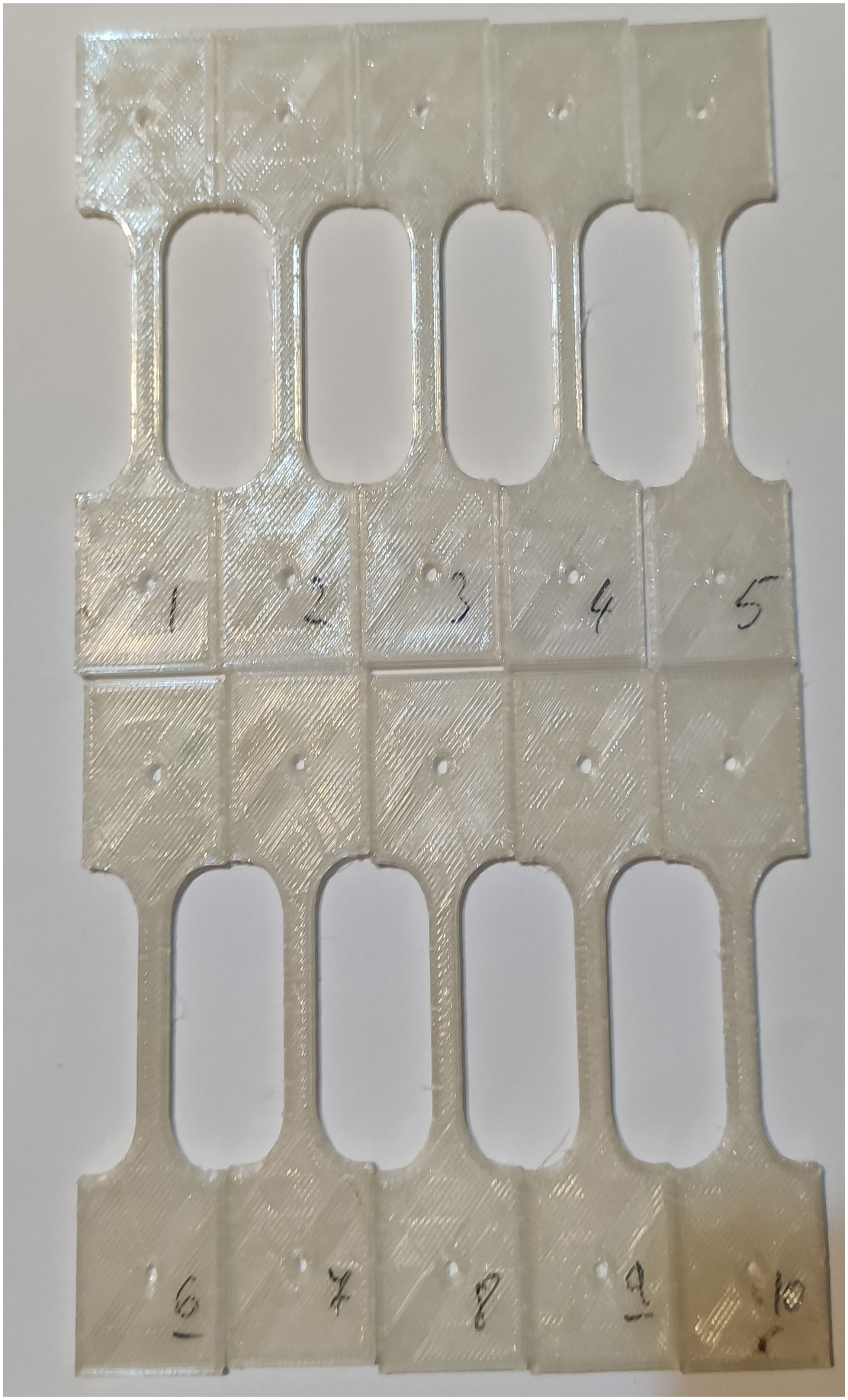

Figure S4. 3D-printed parts for tensile creep testing, fromPETG.

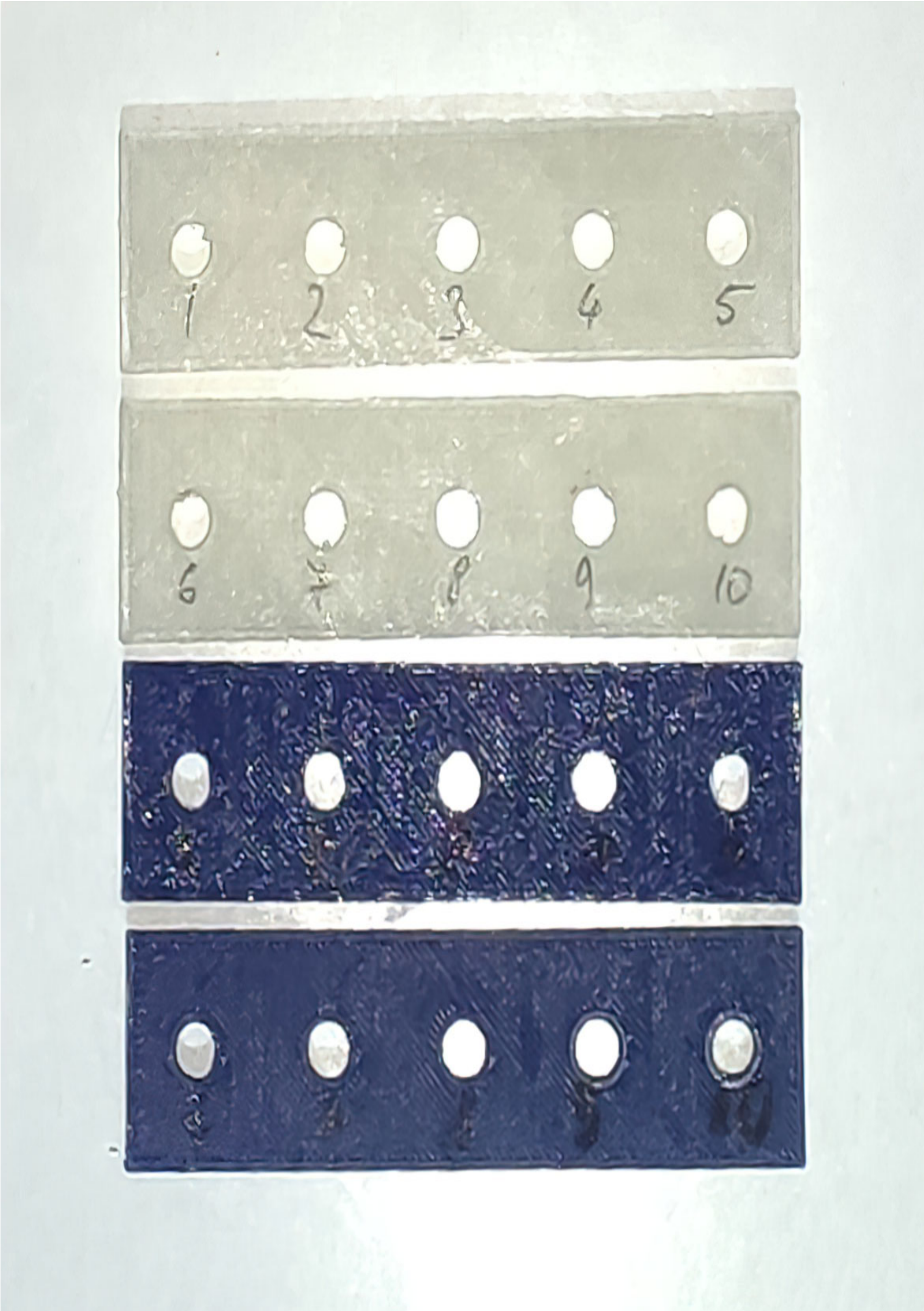

Figure S5. 3D-printed parts for practical compressive creep test, from PLA (blue) and PETG (transparent, white).

Table S1. Dimensional measurements.

	Tensile strength		Compression strength			Creep (tensile)		Creep (compress)	
	**Width**	**Height**	**X**	**Y**	**Z**	**Width**	**Height**	**Y**	**Z**
**Nominal [mm]**	13.00	4.00	15.00	15.00	15.00	5.20	3.00	25.20	8.00
**PLA Control [mm]**	13.292 ± 0.015	4.016 ± 0.009	15.602 ± 0.022	15.316 ± 0.011	15.018 ± 0.009	5.204 ± 0.006	3.000 ± 0.004	25.188 ± 0.006	8.076 ± 0.013
**PLA UV-B [mm]**	13.268 ± 0.030	4.010 ± 0.010	15.588 ± 0.029	15.324 ± 0.007	15.034 ± 0.010	5.212 ± 0.005	3.004 ± 0.005	25.196 ± 0.007	8.092 ± 0.017
**PETG Control [mm]**	13.336 ± 0.014	4.020 ± 0.005	15.648 ± 0.020	15.428 ± 0.010	15.016 ± 0.005	5.210 ± 0.008	3.014 ± 0.005	25.280 ± 0.012	7.604 ± 0.004
**PETG UV-B [mm]**	13.360 ± 0.014	4.016 ± 0.005	15.770 ± 0.046	15.446 ± 0.013	15.020 ± 0.010	5.210 ± 0.004	3.012 ± 0.006	25.300 ± 0.013	7.604 ± 0.005
